# Prevalence of alexithymia and depression among professional contemporary French dancers

**DOI:** 10.3389/fspor.2025.1515051

**Published:** 2025-04-10

**Authors:** Catarina Proenca Lopes, Mathias Poussel, Eliane Albuisson, Margaux Temperelli, Oriane Hily, Bruno Chenuel, Edem Allado

**Affiliations:** ^1^ Université de Lorraine, DevAH, Nancy, France; ^2^ Université de Lorraine, CHRU-Nancy, University Center of Sports Medicine and Adapted Physical Activity, Nancy, France; ^3^ CHRU-Nancy, Direction de la Recherche Clinique et de l’Innovation, Nancy, France; ^4^ Université de Lorraine, CNRS, IECL, Nancy, France; ^5^ Université de Lorraine, Département du Grand Est de Recherche en Soins Primaires: DEGERESP, Nancy, France

**Keywords:** alexithymia, depression, anxiety, contemporary dancers, physical activity

## Abstract

**Background:**

A high incidence of mental disorders has been observed in contemporary dance, characterized by the integration of physical performances with artistic demands. The aim of this study is to evaluate the prevalence of alexithymia and anxiety in a sample of professional ballet dancers.

**Methods:**

The participants, involved in a national contemporary dance company (“Ballet de Lorraine”), underwent a medical assessment including an exercise test and completed computerized questionnaires on anthropometric data and characteristics of sports practice (level and weekly time spent on sports practice) as well as alexithymia (TAS 20), depression (BDI-13), and anxiety traits (STAI-Y form B).

**Results:**

A total of 22 professional dancers were included. This study revealed a prevalence of alexithymia at 9.1%. Within the same sample, the prevalence of anxiety was 9.1%, and 7 dancers showed results indicative of minor depression.

**Conclusion:**

The prevalence of alexithymia, depression, and anxiety is lower in the population of professional contemporary dancers compared to what has been reported in the general and athletic populations. This may be attributed to the artistic intention present in contemporary dance, which might enhance emotional awareness and expression, potentially acting as a protective factor against these psychological conditions. However, further studies with larger and more diverse samples are necessary to confirm these findings and to explore the underlying mechanisms.

## Introduction

Alexithymia has recently been recognized as a personality trait present in the general population (1, 2). Since it was first mentioned at a European psychosomatic research conference in 1970, alexithymia has been defined as an inability to recognize and describe emotions, the use of concrete words and thoughts related to external events, and a scarcity of imaginative life (1, 3). The alexithymia prevalence in the general population ranges from 8% to 23% (4).

Studies have demonstrated the association between alexithymia, anxiety, and depression in the general population (5–10). These studies have shown that alexithymia is linked to heightened levels of anxiety and depression in the general population. The primary hypothesis is that symptoms of anxiety and depression may impede a person's ability to learn how to recognize and express their emotions, thereby intensifying the challenges associated with alexithymia. Although it has been established that sports are a protective factor against anxiety and depression, some studies advocate for incorporating exercise as a supplement to medication in individuals with treatment-resistant depression (11). Sports are also strongly associated with alexithymia, especially in some high-risk sports. The high-risk environment is central to studies on alexithymia in sports. Theoretical perspectives suggest that such an environment may facilitate emotion regulation, thereby helping individuals to experience and manage anxiety effectively, which can be particularly beneficial (12). Additionally, a dose-effect of the number of training hours has been highlighted, with a higher prevalence of alexithymia among athletes training more than 5 h per week compared to those not exceeding this threshold, being at 34.4% vs. 27.7% (13). These individuals may over-adapt to their environments, becoming unaware of their internal cues (such as physical and emotional exhaustion), which ultimately contributes to a reduced motivation for competition (12). This phenomenon is also observed in the context of competitive preparation compared to recreational practice (13).

Professional dance is characterized by the integration of physical performances with artistic demands, clearly setting it apart from purely sporting or artistic activities. This specificity exposes professional dancers to high levels of stress and anxiety in their careers. Among the stress factors are the high expectations related to the pursuit of artistic perfection, weight constraints, intensive training programs, fierce competition, and notable job insecurity (14). Thus, the multiple risks associated with the practice of dance can make it a significant factor contributing to alexithymia.

This study aims to evaluate the prevalence of alexithymia within a company of professional contemporary dancers. It also intends to assess the maximal functional capacities under effort of this population.

## Methods

We performed a transversal, monocentric study at the University Center of Sports Medicine and Adapted Physical Activity, University Hospital of Nancy, France, from 01/08/2020 to 01/05/2021 in a sample of professional dancers from a contemporary national company (Centre Chorégraphique National—Ballet de Lorraine) during annual medical pre-season assessment. This center is a recognized expertise hub for monitoring professional athletes and high-level sports performers. It has been overseeing the dance company since 2018. The different inclusion criteria were age, over 18 years old, and more than 10 years of dance practice. All dancers in the company are professional dancers with a dance practice exceeding 35 h per week.

### Demographic clinical data and intervention

The data collected from the patients included age, gender, Body Mass Index (BMI), resting heart rate (HRrest) measured via electrocardiogram (ECG). and cardiopulmonary exercise test.

The study also included 3 self-administered questionnaires to evaluate different psychological aspects. These instruments were selected for their widespread use in the field and strong psychometric properties. Additionally, they were quick (5–10 min) and easy to complete, which was crucial since the questionnaires were completed by the participants independently.

1. The Toronto Alexithymia Scale (TAS-20) consists of 3 subscales that combine to yield a total alexithymia score, where higher scores indicate higher levels of alexithymia (15). A score of 46–55 suggests a “borderline” status, while a score of 56 or above indicates the presence of alexithymia. The 3 subscales are: difficulty identifying feelings (DIF) with 7 items, difficulty describing feelings (DDF) with 5 items, and externally oriented thinking (EOT) with 8 items.
2. Beck's Short Depression Inventory (BDI-13) assesses the severity of depression with 13 items, ranging from 0 to 39. A score of 4 indicates mild depression, scores above 8 indicate moderate depression, and scores above 16 indicate severe depression (16).
3. The Spielberger State–Trait Anxiety Inventory (STAI) measures anxiety levels (17). It distinguishes state anxiety (State Anxiety—STAI-S) and trait anxiety (Trait Anxiety—STAI-T). Each is measured with a 20-item scale ranging from 20 to 80. Anxiety scores above 56 are high, and above 65 are very high.

All of the questionnaires have been translated and validated in French (18, 19).

### Statistical analysis

Both descriptive and comparative analyses were conducted by accounting for the nature and distribution of the variables. Qualitative variables were described as frequencies and percentages; quantitative variables were evaluated with the mean ± standard deviation (SD) or with the median and interquartile range (IQR). The significance level was set at 0.05 for the entire study. IBM™ SPSS Statistics v23 was used for the data analysis.

### Ethics and dissemination

All data used were extracted from the medical records. No additional examination was necessary for patients to meet the inclusion criteria. This study was designed in accordance with the general ethical principles outlined in the Declaration of Helsinki. The protocol of this study was approved by the Information Technology and Freedoms Commission. All patients gave their verbal consent for the use of their medical data during their medical care at the University Hospital.

## Results

### Demographic, clinical data and intervention

In our study, all members of the national contemporary dance company were included (22 people). Eleven (50.0%) were women with a mean age of 27.0 (±6.5) years, a mean BMI of 20.5 (±1.8), a mean HR_rest_ 69.1 (±12.6) bpm (Table 1).

**Table 1:** Baseline demographic, clinical and psychological, characteristics (*n* = 22).

Characteristics	Total (*n* = 22)
Women	11 (50.0)
Age, years	27.0 (±6.5)
Body mass index, kg/m^2^	20.5 (±1.8)
– Height	170.6 (±8.4)
– Weight	60.0 (±9.0)
HR_rest_	69.1 (±12.6)
STAI-S (State Anxiety)	31.9 (±7.5)
STAI-T (Trait Anxiety)	39.5 (±7.5)
TAS 20 (Alexithymia)	41.6 (±5.8)
BDI 13 (Depression)	2.6 (±2.1)

Data are presented as *n* (%) for dichotomous variables, mean (±SD) for continuous demographic variables with normal distribution and median [interquartile range] with non-normal distribution.

### Psychological aspects

The prevalence of alexithymia, depression, and anxiety among the participants is presented in Table 1. According to the TAS 20, 16 participants (72.7%) had no alexithymia (scores <46), 4 participants (18.2%) were classified as borderline (scores 46–55), and 2 participants (9.1%) had alexithymia (scores >55). For depression (using the BDI), 17 participants (77.3%) showed no signs of depression, 3 participants (13.6%) exhibited mild depression, and 2 participants (9.1%) had moderate depression. Anxiety (assessed with the STAI), revealed that 2 participants (9.1%) experienced severe or very high levels of anxiety (scores >56). Notably, only one dancer exhibited both anxiety and alexithymia (Table 2).

**Table 2:** Psychological aspect per dancers (anxiety, alexithymia and depression) (*n* = 22).

Dancer	State Anxiety STAI-S	Trait Anxiety STAI-T	TAS 20	BDI 13
1	27	36	35	1
2	26	38	37	2
3	26	26	39	1
4	24	38	32	0
5	40	59	47	8
6	34	41	34	2
7	37	43	44	3
8	27	47	40	1
9	28	34	41	1
10	47	49	40	5
11	63	55	56	4
12	NA	NA	39	9
13	21	36	32	1
14	27	25	39	0
15	27	29	37	0
16	21	28	47	0
17	36	38	33	4
18	37	47	45	3
19	27	51	49	5
20	39	35	43	1
21	34	32	50	1
22	22	42	57	5

Toronto Alexithymia Scale (TAS-20): [20; 45]: no alexythimia, [46; 55]: borderline status, [56; 100]: alexithymia; Beck's Short Depression Inventory (BDI-13) [4; 7]: mild depression, [8; 15]: moderate depression, and [16; 39]: severe depression; State–Trait Anxiety Inventory (STAI), for each [20; 55]: no anxiety, [56; 65]: high anxiety and [66; 80] very high anxiety, NA: not available.

## Discussion

This study, conducted with a national professional contemporary dance company, revealed a prevalence of alexithymia at 9.1%. Within the same sample, the prevalence of anxiety was 9.1%, and 7 dancers showed results indicative of minor depression.

The alexithymia prevalence in the general population ranges from 8% to 27% and from 23% to 40% in the athletes (4). The results of our study revealed 2 individuals with alexithymia, one in association with anxiety, and none with associated depressive states. A strong association between alexithymia, anxiety, and depression has nevertheless been demonstrated (20–22). Thus, we can an effective alexithymia prevalence of 4.6% (*n* = 1) in this study, which is significantly lower than what is reported in the literature. Although the maximal exercise capacity of the professional contemporary dancers in our sample are equivalent to those observed in high-level athletes (23, 24), we found a significantly lower prevalence of alexithymia. This may be explained by the artistic intent present in contemporary dance, which differs from the sole performance focus of high-level sports and classical dance.

Several studies have highlighted a strong association of psychological disorders, such as eating disorders, with a prevalence of 16.4% among ballet dancers compared to 12.0% in the general population, as well as anxiety and depression among professional dancers (14, 25–27). An increased presence of addictive disorders has also been observed in this population (14). These disorders show a notable association with alexithymia, with prevalence rates for psychosomatic disorders, depression, and alcoholism/substance abuse of 30%–60%, 50%, and 40%–80%, respectively (4, 28, 29). However, although this study did not record the presence of addictions, as it was conducted within a routine care setting, the low prevalence of alexithymic individuals observed is reassuring. This suggests that dance could serve as a tool for emotional expression. As in the study by Bojner et al., the low presence of alexithymia in our study could be explained by a more developed awareness of emotional processing and interpretation among dancers (30). Dance allows strong emotional communication with others. Thus, although the subjects are in the field of performance, ballet, like any other type of dance, aims to convey emotional experiences, unlike non-artistic sports, which function as emotional regulators for alexithymic athletes through bodily overstimulation (sensation-seeking). Therefore, dance may promote the development and understanding of emotional qualities, as suggested by Behrends' study on empathy (31). In this sense, a study highlighted a positive effect of rhythmic movement therapy on alexithymia (32).

The limitations of this study lie in its monocentric nature. By focusing on a single contemporary dance company, representing a specific type of dance, these results cannot be generalized to all types of practice. Although the small sample size of the study may appear to be a weakness, the inclusion of one of the three national contemporary dance companies with a practice time greater than 35 h per week is a strength. Additionally, the absence of data collection on eating or addictive disorders within this population represents another limitation. Then the study was conducted during the COVID-19 pandemic, which may have impacted the psychological state of the dancers. However, the fact that this study focuses on a professional company allows for the control of extrinsic factors specific to artistic/sporting practice (hours of training, tours, number of performances per year). Furthermore, conducting effort tests for the entire population allows for the evaluation of the physiological capacities under effort of this population and confirms its comparability with that of high-level athletes.

In conclusion, this study, conducted on a population of professional dancers from a national contemporary dance company, observed a lower prevalence of alexithymia and anxiety compared to the general population and to what has been described in the literature for athletes. These results may suggest a protective effect of physical activities aimed at artistic expression against alexithymia. However, it will be necessary to conduct similar studies on a larger population and from multiple professional companies to confirm these observations.

## Data Availability

The raw data supporting the conclusions of this article will be made available by the authors, without undue reservation.
